# Supplementary figures and images for: Mesenchymal stem cell-derived exosome and liposome hybrids as transfection nanocarriers of Cas9-GFP plasmid to HEK293T cells
Source: PLoS One. 2025 Jan 13;20(1):e0315168. doi: 10.1371/journal.pone.0315168 (PMC11729927; doi:10.1371/journal.pone.0315168)

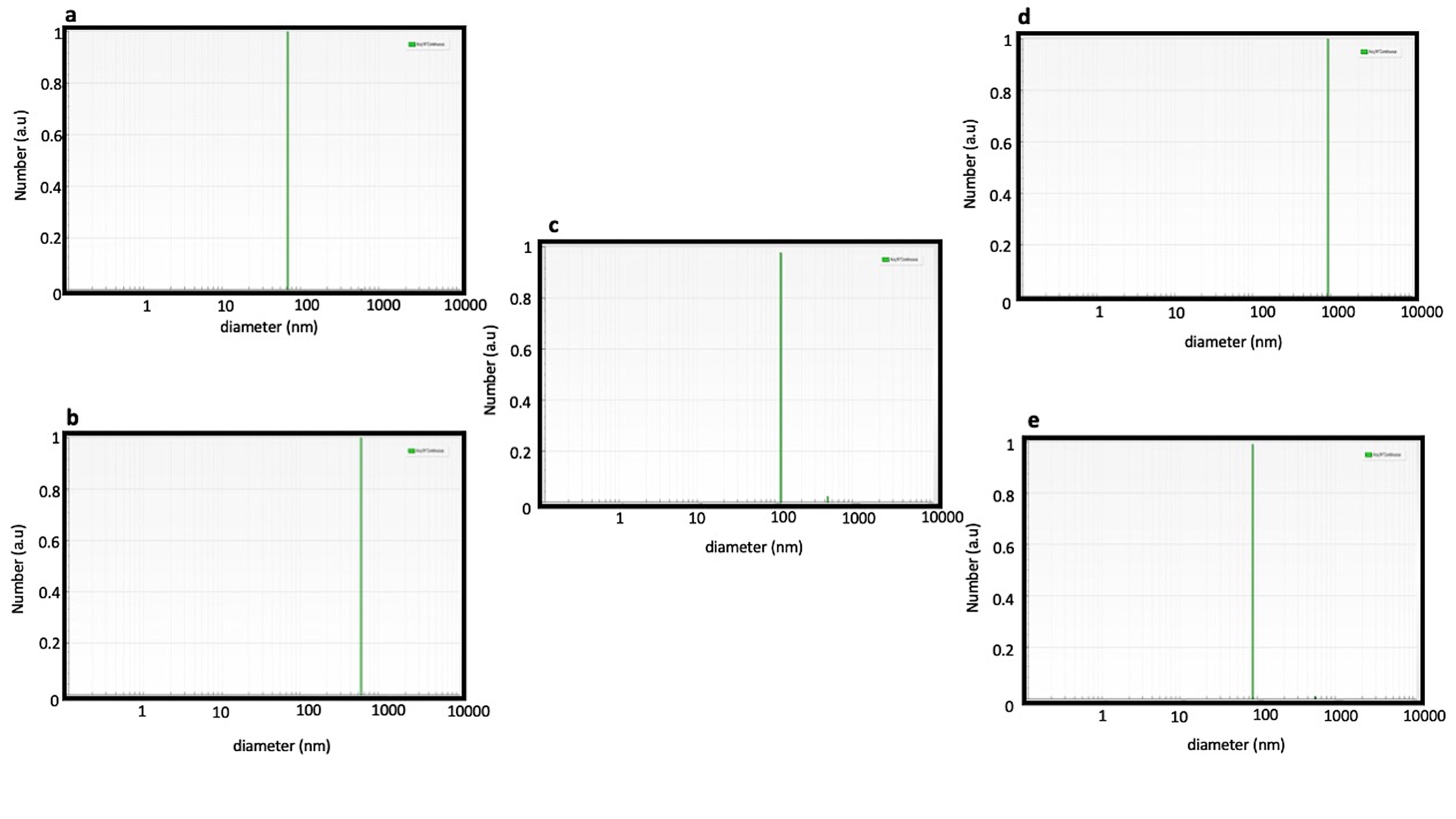

Supplement: S1 Fig — a) 63.1 nm (fractions 6–10), b) 540.63 nm (sample 1), c) 108.98 nm (sample 2), d) 827.21 nm (sample 3), e) 76.72 nm (sample 4). (TIF) [file pone.0315168.s001.tif]

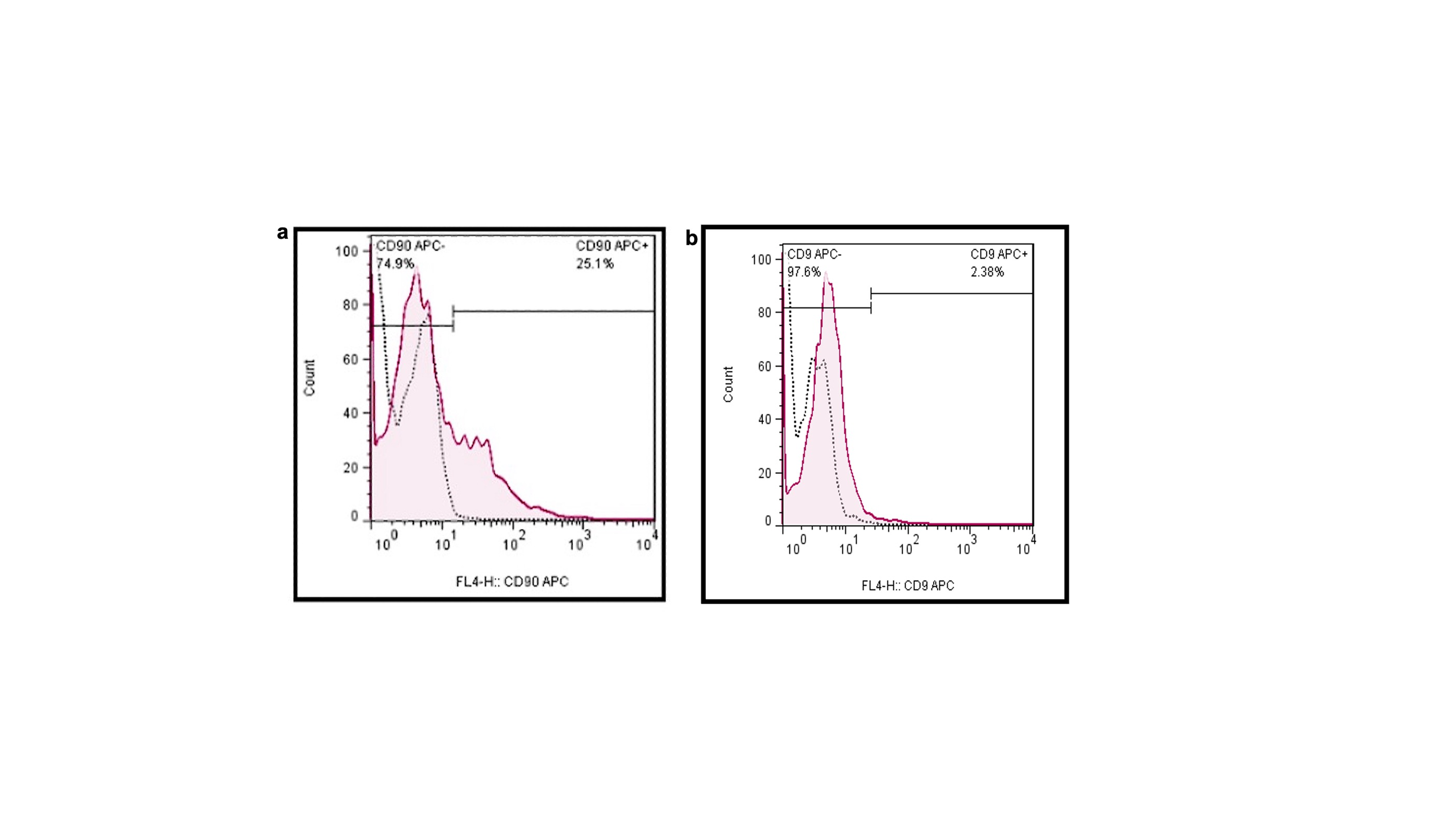

Supplement: S2 Fig — BM-hMSCs-Derived Exosome Fractions 1–9 Characterized for a) CD90, and b) CD9 Markers, Showing 25.1% CD90 and 2.3% CD9 Expression. (TIF) [file pone.0315168.s002.tif]

1 2 3 4 5 6 7 8

100

63

48

35

25

12

11

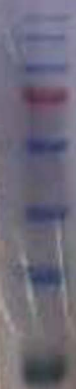

L 9 10 11 12 13 14 15 16 17 18 19

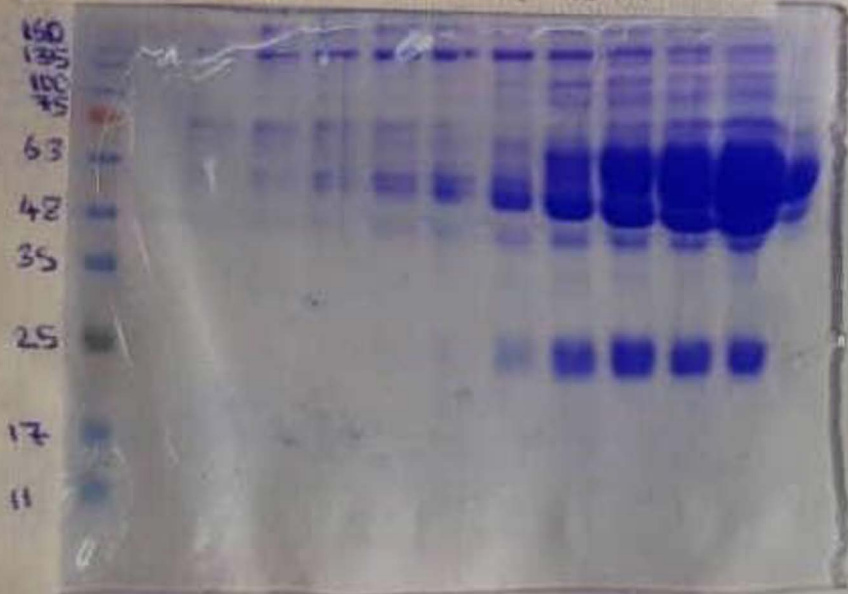

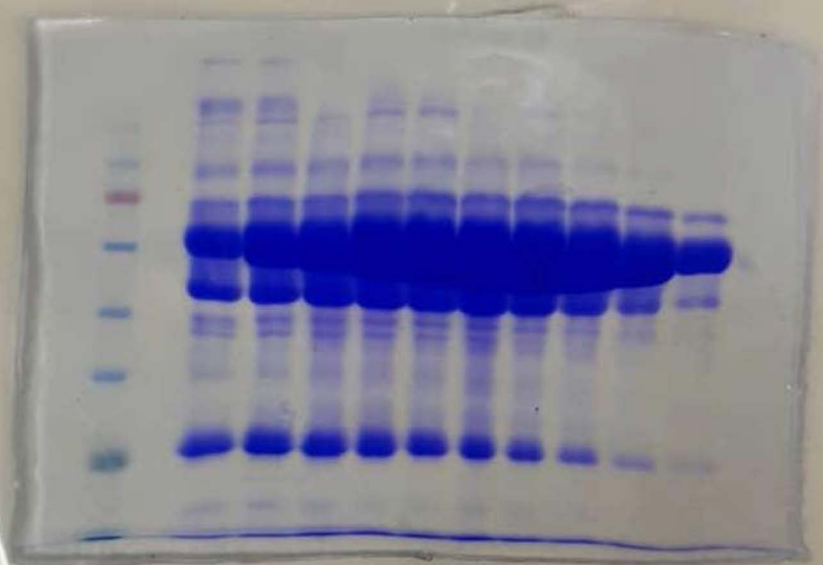

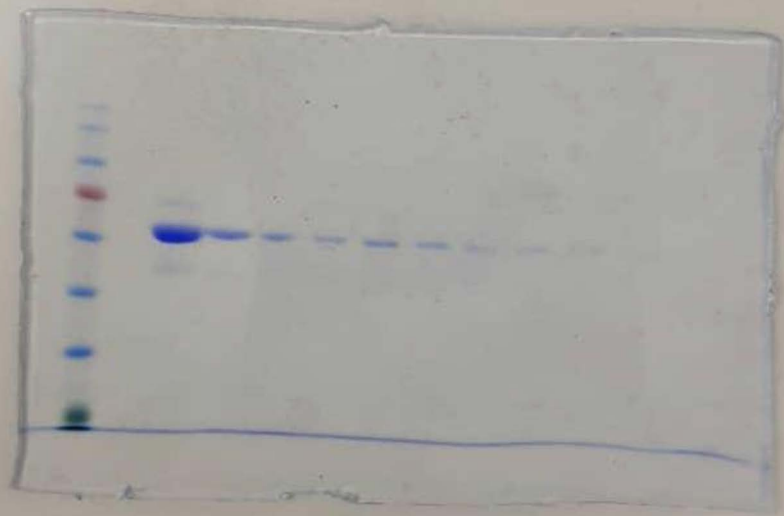

Supplement: S1 Raw images — (PDF) [file pone.0315168.s003.pdf]
